# Helping Babies Breathe, Second Edition: A Model for Strengthening Educational Programs to Increase Global Newborn Survival

**DOI:** 10.9745/GHSP-D-18-00147

**Published:** 2018-10-03

**Authors:** Beena D. Kamath-Rayne, Anu Thukral, Michael K. Visick, Eileen Schoen, Erick Amick, Ashok Deorari, Carrie Jo Cain, William J. Keenan, Nalini Singhal, George A. Little, Susan Niermeyer

**Affiliations:** a Department of Pediatrics, University of Cincinnati College of Medicine, Cincinnati, OH, USA, and Perinatal Institute and Global Child Health, Cincinnati Children's Hospital Medical Center, Cincinnati, OH, USA.; b Department of Pediatrics, All India Institute of Medical Sciences, New Delhi, India.; c Latter-day Saint Charities, Salt Lake City, UT, USA.; d Division of Life Support, American Academy of Pediatrics, Itasca, IL, USA.; e World Hope International, Alexandria, VA, USA and Freetown, Sierra Leone.; f Division of Neonatology, Saint Louis University, St. Louis, MO, USA.; g Division of Neonatology, University of Calgary, Alberta, Canada.; h Division of Neonatology, Geisel School of Medicine at Dartmouth, Hanover, NH, USA.; i Section of Neonatology, University of Colorado School of Medicine, Aurora, CO, USA.

## Abstract

**Background:**

Helping Babies Breathe (HBB), a skills-based program in neonatal resuscitation for birth attendants in resource-limited settings, has been implemented in over 80 countries since 2010. Implementation studies of HBB incorporating low-dose high-frequency practice and quality improvement show substantial reductions in fresh stillbirth and first-day neonatal mortality. Revision of the program aimed to further augment provider and facilitator skills and address gaps in implementation with the goal of improving neonatal survival.

**Methods:**

The Utstein Formula for Survival—Medical Science X Educational Efficiency X Local Implementation = Survival—provided a framework for the revisions. The 2015 Neonatal Resuscitation Consensus on Science and Treatment Recommendations by the International Liaison Committee on Resuscitation informed scientific updates, which were harmonized with the 2012 World Health Organization Basic Newborn Resuscitation Guidelines. Published literature and program reports, consensus guidelines on reprocessing equipment, systematic collection of suggestions from frontline users, and responses to a semistructured online questionnaire informed educational/implementation revisions. Links to maternal care were added. Draft materials underwent Delphi review and field testing in India and Sierra Leone. An Utstein-style meeting of stakeholders identified key actions for successful implementation.

**Results:**

Scientific revisions included expectant management of infants with meconium-stained amniotic fluid, limitation of suctioning, and initiating and continuing effective ventilation until spontaneous respirations. Frontline users (N=102) suggested augmented simulation methods to build confidence and competence and additional guidance for facilitators on implementation. Users identified a need for sufficient practice during the workshop, systematized ongoing practice, and enough simulators for participants. Field trials refined approaches to self-reflection, feedback and debriefing, and quality improvement. Utstein meeting stakeholders validated the importance of quality improvement and use of data to improve outcomes.

**Conclusions:**

The second edition of HBB provides a newer paradigm of learning for providers that incorporates workshop practice, self-reflection, and feedback and debriefing to reinforce learning as well as the promotion of mentorship and development of facilitators, systems for low-dose high-frequency practice in facilities, and quality improvement related to neonatal resuscitation.

## Abstract (precis)

The revised neonatal resuscitation curriculum updates not only the science of resuscitation but also the educational and implementation approaches needed to further enhance neonatal survival, including promoting ongoing practice to retain skills and linkages with quality improvement initiatives.

## INTRODUCTION

Intrapartum-related events—also known as birth asphyxia—occur between the beginning of labor and the delivery of the placenta; they are a major cause of neonatal morbidity and mortality and the primary cause of intrapartum stillbirths.^1^^,^^2^ Although neonatal resuscitation is an intervention that has the potential to save newborn lives and reduce injury,^3^^–^^5^ widespread and effective implementation has been challenging. Helping Babies Breathe (HBB) is a global curriculum for neonatal resuscitation specifically designed to simplify and demystify the resuscitation steps.^6^^–^^8^ The skills-based curriculum focuses on enhancing birth attendants' understanding and basic resuscitation skills through active learning with simulation, emphasizing practice with peers to develop teamwork, good communication, and reflective learning with self-improvement. Indeed, HBB challenges the previous assumption that equated resuscitation with neonatal intensive care and instead promotes the idea that basic neonatal resuscitation should be available to every baby, wherever they are born.

The first edition of the HBB curriculum was developed by the Global Implementation Task Force, which was founded in 2006 and consisted of stakeholders brought together by the American Academy of Pediatrics (AAP) to develop a standardized, simplified neonatal resuscitation curriculum based on the same evidence as the Neonatal Resuscitation Program.^8^^–^^11^ Informed by global expertise in education and neonatal care, the resulting educational program focused on active learning with simulation and pictorial materials. The curriculum, designed to harmonize with the World Health Organization (WHO) Basic Newborn Resuscitation Guidelines (then under revision) and the 2010 Consensus on Science and Treatment Recommendations (CoSTR) by the International Liaison Committee on Resuscitation (ILCOR),^12^^,^^13^ underwent 2 rounds of Delphi review to build consensus between qualified external experts.^14^ It was then field tested in Bangladesh, India, Kenya, Pakistan, and Tanzania before being revised and released.^9^^,^^10^^,^^15^

In 2010, a public-private partnership, the HBB Global Development Alliance (GDA), was created. The 5 founding member organizations—AAP, Laerdal, National Institute of Child Health and Human Development, Save the Children, and the United States Agency for International Development (USAID)—believed that by working together they could help reduce neonatal morbidity and mortality. These educational and neonatal care experts began to design a curriculum, develop implementation plans, and coordinate training efforts for an educational program to strengthen the knowledge and skills of birth attendants who care for mothers and babies in low-resource settings.^9^ Since rollout of the program in 2010, HBB workshops have taken place in more than 80 countries—with the curriculum translated into 27 languages—and an estimated 500,000 providers trained.^9^^,^^16^ Before and after studies of regional or facility-based HBB training in Africa and Asia have shown substantial decreases in very early neonatal mortality, stillbirth rates, and asphyxia-related morbidity and mortality when provider education was coupled with facilitated ongoing practice, quality improvement assessments, and local ownership of the program that integrated the content into routine clinical practice.^17^^–^^20^

Since rollout of the Helping Babies Breathe program in 2010, workshops have taken place in >80 countries, with an estimated 500,000 providers trained.

While these successes were celebrated, analysis of the published literature and the experience of implementing partners of the HBB GDA highlighted elements of the educational package and implementation approach that needed strengthening. Without systematic, integrated, and sustained activities, the trainings by themselves were unlikely to result in longstanding change,^11^^,^^16^^,^^21^ and the first edition of the HBB curriculum did not contain guidance on these issues. For example, case reports describing the implementation of the HBB curriculum in Bangladesh and Malawi demonstrated improvements in the provision of neonatal resuscitation, but a lack of improvement in neonatal mortality when the program was implemented widely, but incompletely, without a plan for ongoing exposure, practice, and quality improvement efforts.^11^^,^^22^ Conversely, concerted efforts to include ongoing practice and quality improvement assessments in studies performed in Africa and Asia demonstrated further reductions in neonatal mortality after HBB training.^18^^,^^20^^,^^23^ Lessons learned during the first 5 years of program implementation indicated that adaptation of materials for local contexts must be facilitated and systematic ongoing practice—extending beyond the duration of a training workshop—should be embraced.^18^^,^^24^^,^^25^ Furthermore, achieving impact at the population level requires integration of the curriculum into the regional health system, with integration of adapted educational materials into comprehensive preservice and in-service education packages, mechanisms for supply and logistics management, and linkages with ongoing quality improvement initiatives to effect change and document outcomes.

Evolving evidence and further acknowledgment of the challenges of implementation and sustainability were incorporated into the second edition of the HBB curriculum and are outlined here. The goals of this process were to further improve neonatal care by promoting the most current science, augmenting educational effectiveness, and suggesting expanded implementation strategies. Through documenting the process by which the inputs for revision were incorporated into the new edition, we intend to provide a model for continuous improvement of perinatal education programs.

The second edition of the Helping Babies Breathe curriculum aimed to further improve neonatal care by promoting the most current science, augmenting educational effectiveness, and suggesting expanded implementation strategies.

## METHODS

### Inputs for Revisions

**FIGURE 1:**
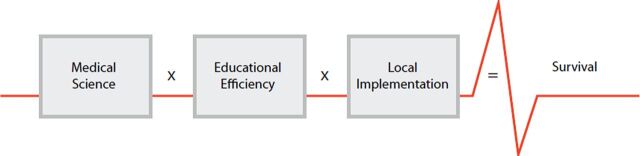
The Utstein Formula of Survival Adapted with permission from Søreide et al.^27^

In 2015, an Utstein-style meeting of key stakeholders focused on previous implementation of the HBB curriculum to determine what key actions were essential for effective dissemination of educational programs for neonatal and maternal survival, such as the Helping Babies Survive and Helping Mothers Survive programs. The framework for improving survival worldwide is summarized in the Utstein Formula for Survival, based on the consensus of international experts, which states that survival is the product of medical science, educational effectiveness, and implementation (Figure 1).^26^^,^^27^ Although the development of the first edition of the HBB curriculum focused on the design of the educational program, adding the components of the Utstein Formula for Survival to the second edition helped provide a framework for identifying changes that resulted from an additional focus on enhanced educational effectiveness, skills retention, and the importance of coordination with national resources and leadership. The framework also identified 2 key challenges: sustainability and wide implementation. The inputs that aided the revisions are described in further detail below.

#### Resuscitation Science

The goal of the HBB curriculum is to bring the latest in resuscitation science to low-resource settings. To that end, the 2015 ILCOR CoSTR was formed to provide a system for evaluating scientific updates.^28^ For the first time, the 2015 ILCOR CoSTR used the Grading of Recommendations, Assessment, Development, and Evaluations (GRADE) approach for evaluating evidence to rate guidelines recommendations, based on the strength of the evidence.^29^ The most recent changes in resuscitation processes identified by the 2015 ILCOR CoSTR review were further harmonized with the revised WHO *Guidelines on Basic Newborn Resuscitation*.^12^

During the evaluation process, the committee also reviewed the evidence supporting delayed cord clamping. New experimental evidence and human data from low-resource settings demonstrated increased neonatal morbidity and mortality with cord clamping prior to onset of respiration.^30^^–^^32^ Feedback from user experience revealed a frequent overreliance on suctioning, whether the infant was breathing or not; delays in the initiation of ventilation; and frequent interruptions in ventilation when the infant was not yet breathing.

The committee also recognized that a single provider may often be caring for the mother–infant pair and that little evidence was available on how to co-manage the 2 patients if both were critically ill. Improved linkages between neonatal care and maternal care were made, including, for example, the preparation of oxytocin before birth.

Researchers in Kenya expressed concern that improper or incomplete disinfection of resuscitation equipment was a contributing factor in spreading infection.^33^ They reported that non-HBB-trained personnel were often involved in reprocessing the equipment for future use, which was being done improperly, potentially affecting the safety and functionality of the equipment.^33^ They noted that a workable field guide did not exist that would provide recommendations about reprocessing of used resuscitation equipment.

#### Educational Effectiveness

The dissemination of the HBB curriculum, as noted earlier, was global, with numerous facility-based studies of doctors, nurses, and midwives indicating that uptake of both knowledge and skills improved immediately after an HBB workshop.^15^^,^^24^^,^^34^^–^^36^ However, published reports also mentioned the deterioration of skills after HBB workshops, which mirrored the experiences of other resuscitation training programs.^24^^,^^37^^,^^38^ For the effective performance of these lifesaving skills to impact neonatal mortality and stillbirth rates, providers need to be able to perform basic resuscitation and bag-mask ventilation, if needed, within “The Golden Minute” after birth.

Numerous studies since the release of the first edition of the curriculum indicate that a system of ongoing practice or refresher training can be effective for the maintenance of resuscitation skills.^39^ Many key lifesaving skills, such as bag-mask ventilation, require more practice time, focus, and supervision than could be provided during the usual 1-day workshop.^24^ While the exact frequency of practice and refresher training required to maintain proficiency for each type of provider is unknown, it is clear that ongoing low-dose high-frequency practice can improve performance and competency.^18^^,^^23^^,^^25^^,^^40^^,^^41^ Importantly, the incorporation of debriefings and case reviews after real-life delivery room situations, and a quick review of bag-mask ventilation in low-dose high-frequency sessions, for example, at the beginning of a shift, improved early neonatal mortality and decreased stillbirth rates in facility-based settings in Africa and Asia.^18^^,^^23^

**Figure:**
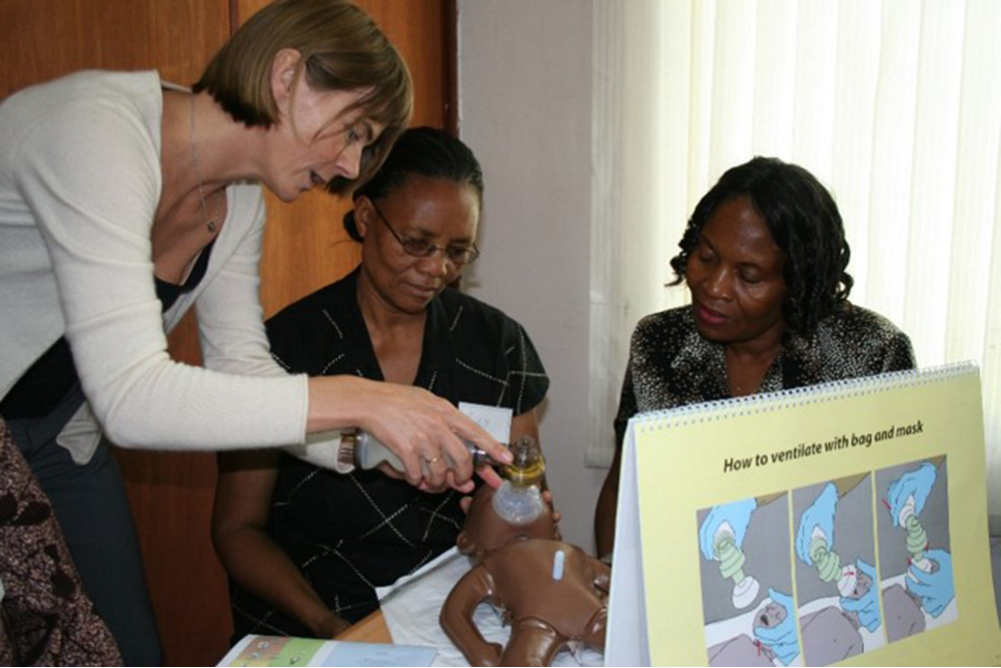
Pilot testing of Helping Babies Breathe 1st Edition in Dar es Salaam, Tanzania. © 2010 Eileen Schoen/American Academy of Pediatrics.

Lifesaving skills, such as bag-mask ventilation, often require more practice time, focus, and supervision than can be provided in a single-day workshop.

Studies also indicated that it was important to consider past experience of the providers, as different cadres of providers such as physicians likely had some past experience with neonatal resuscitation training and simulation, whereas nurses did not.^41^ Furthermore, researchers noted differences between who was able to perform these skills in real-life scenarios, despite similar performances during simulation exercises. The concept of ongoing practice, even when studied in rural providers—such as village midwives and birth attendants—1 year after their initial HBB training, showed retention of basic resuscitation skills with ongoing practice and/or refresher trainings and reductions in fresh stillbirth and early neonatal mortality rates.^38^^,^^42^^,^^43^

Finally, additional input from frontline users also noted that the skills assessments—in particular, the objective structured clinical evaluations (OSCEs)—were cumbersome, confusing, and potentially biased. These assessments were often used in both summative and formative evaluation but were not always implemented in a learner-focused fashion, which allows learners to self-reflect and learn from their experience.

#### Implementation and Sustainability

After gathering information from published literature and program reports, the HBB GDA published a summary of the first 5 years of HBB implementation, with a clear message that gaps in quality of care would need to be overcome by more than just additional or continued provider training.^11^ To that end, USAID and WHO designed frameworks for characterizing gaps in quality of care for mothers and babies and strategies to overcome the gaps in care.^44^^,^^45^ The WHO framework described 6 strategic areas where evidence-based approaches could guide interventions to improve care, including the development of clinical guidelines, standards of care, effective interventions, measures of quality of care, relevant research, and capacity-building practices.^45^ The newly formed Quality of Care Network, linked to the WHO framework, focuses on the tenets of quality, equity, and dignity to drive quality of care and access to care for all.

Themes of effective implementation included linking workshops to existing health care programs and leaders in order to promote local ownership and planning for training-of-trainers cascades, with an emphasis on early exposure through preservice education. The Utstein-style meeting formulated 10 essential action points for national dissemination and implementation of the Helping Babies Survive and Helping Mothers Survive program materials and training (Box).

BOX **Essential Action Points for National Helping Babies Breathe and Helping Mothers Survive Implementation**

1. At the country level, establish a maternal, newborn, and child health alliance with public, private, and nongovernmental partners
2. Form a functional working group for advocacy, planning, training, and monitoring at the country level; through the working group, identify gaps in the current system, establish performance standards, set specific goals, and develop a financial plan to implement and sustain the program(s)
3. Develop a plan for nation-to-facility levels training, which achieves high-quality coverage of providers in both public and private facilities
4. Provide appropriately adapted learning materials, equipment, and supplies simultaneously with training
5. Identify and support local leaders and champions
6. Set up local systems for frequent, brief refresher training, debriefing, and audits
7. Support the function of facility-level perinatal quality improvement teams
8. Collect and report local data on a standardized set of indicators of basic processes of care and patient outcomes
9. Develop a system for looped reporting and feedback to/from all levels of the health system and the working group
10. Engage and empower health care providers, famlies, and the broader community in the initiative

Reproduced from Ersdal HL, Singhal N, Msemo G, et al (2017).^26^

To gather additional perspectives from frontline HBB users, we developed a 59-question semistructured online survey. The invitations to participate were sent via email, and the online survey generated 102 responses. The primary respondents were physicians (65%), professionals based in North America (77%), and global HBB facilitators (93%). When asked about the most important change needed to make sure all babies receive help to breathe, respondents answered better confidence and skills in those trained (66%), rather than training greater numbers of providers (33%). When asked about the 3 most important ways to ensure that providers could perform their skills, respondents identified sufficient time for practice during the workshop (91%), enough mannequins to reach the goal ratio of 1 mannequin per 2 participants (54%), and a system for ongoing practice after the workshop (87%). To better support HBB facilitators, respondents ranked facilitating the first course with experienced trainers (68%), improving ways to assess that learners have the required skills (64%), and more instruction/practice on how to facilitate the course (51%) as their 3 highest choices.

Respondents identified sufficient time for practice during workshops and a system for ongoing practice after workshops as key ways to improve provider skills.

#### Delphi Review and Field Trials

The draft materials underwent Delphi review by 20 individuals recruited from frontline users and program managers. Consistent messages from Delphi reviewers included the need to strengthen facilitator advice before, during, and after the workshop; to emphasize systems of ongoing practice and quality improvement after the workshop; and to more strongly link HBB with the Helping Mothers Survive suite of programs. Further inputs from the maternal care community suggested that elements of maternal care could be integrated within HBB, recognizing that care for the mother and baby is often the task of a single provider.

A revised version of the materials underwent field testing in India and Sierra Leone. In India, experienced master trainers, familiar with the first edition materials, and novice participants were trained with the new materials. In Sierra Leone, a group of novice participants was trained to be master trainers, and then observed as they trained a group of providers. At both sites, focus group discussions were performed to obtain qualitative feedback about the new materials and the overall educational program. The interviews were audio recorded, transcribed, and then subjected to thematic analysis by independent reviewers.

### Ethical Considerations

Ethical approval for the semistructured survey was obtained from the Cincinnati Children's Hospital Medical Center Institutional Review Board. For the India field trial, ethical approval was obtained by the Colorado Multiple Institutional Review Board and the Institute Ethics Committee of the All India Institute of Medical Sciences, in New Delhi, India. Ethical approval for the field trial in Sierra Leone was obtained from the Committee for the Protection of Human Subjects at the Theodore Geisel School of Medicine.

## RESULTS

### Resuscitation Science

**FIGURE 2:**
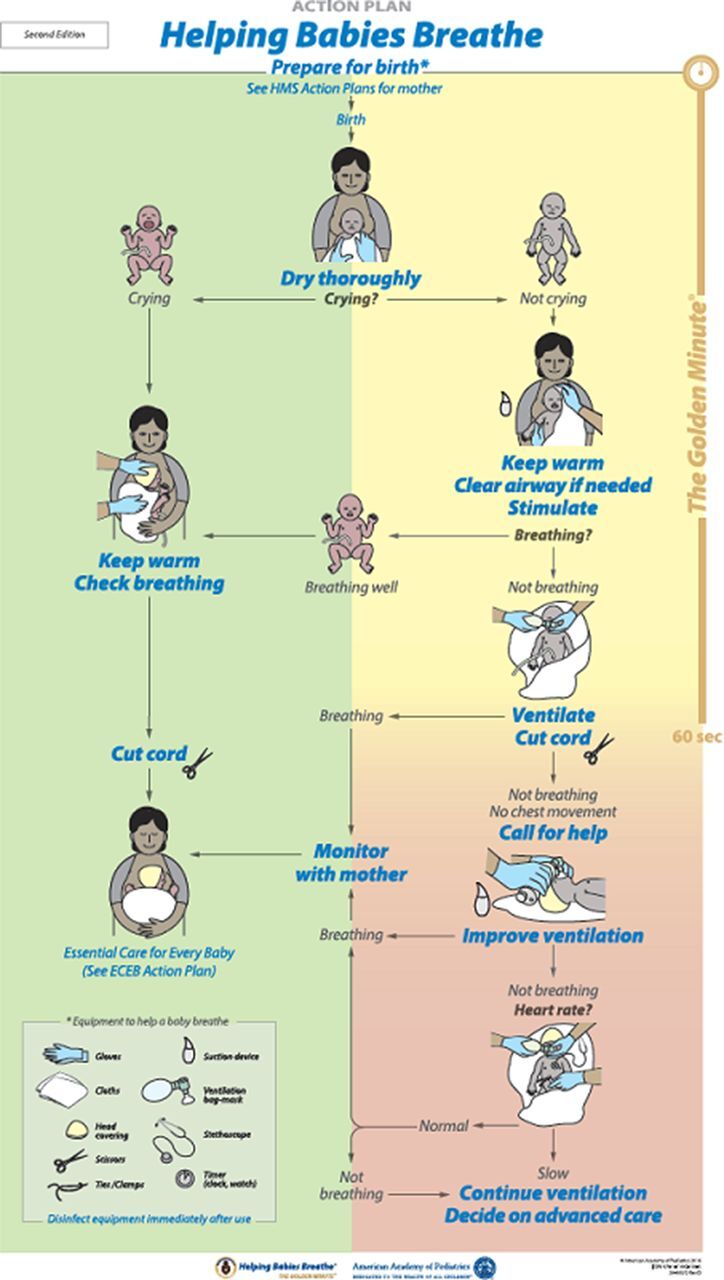
Helping Babies Breathe Second Edition Action Plan Abbreviations: ECEB, Essential Care for Every Baby; HMS, Helping Mothers Survive. Source: Niermeyer S, Kamath-Rayne B, Keenan W, Little G, Singhal N, Visick M, eds. (2016).^7^ Reprinted with permission from the American Academy of Pediatrics.

A summary of the differences between the first and second editions is available on the Helping Babies Survive website (hbs.aap.org) (Supplement).The scientific changes identified in the 2015 ILCOR CoSTR^28^ informed new recommendations in the second edition of the HBB action plan (Figure 2). New recommendations included that no suctioning is needed before drying babies born through meconium-stained amniotic fluid, whether the babies were vigorous or not.^28^ In particular, attention to drying and stimulating the baby after birth resulted in fewer babies requiring bag-mask ventilation.^23^^,^^46^ The second edition further deemphasized the use of oropharyngeal suctioning overall, and stated clearly that it was not needed for infants unless they failed to cry after thorough drying and secretions were seen in the airway. Given the strong evidence for delayed cord clamping, it continued to be incorporated into the second edition action plan (Figure 2). However, a new option was included to initiate ventilation prior to cutting the cord,^30^ with the advice that a facility should determine in advance how they plan to sequence these events, depending on the number of providers at a birth and their ability to ventilate the baby on or by the side of the mother. Similar to the seventh edition of the Neonatal Resuscitation Program, which is also based on the 2015 ILCOR CoSTR, the second edition action plan emphasized providing effective ventilation, with rapid assessment of chest movement and initiation of corrective steps to improve ventilation.^7^

New recommendations focus on drying and stimulating babies immediately after birth regardless of the presence of meconium-stained fluid, which reduces the number of babies requiring bag-mask ventilation.

Finally, to provide recommendations on disinfection and reprocessing of equipment, PATH conducted an evidence-based review of reprocessing basic neonatal resuscitation equipment in resource-limited settings (Supplement).^47^ In the absence of sufficient available evidence, the organization made recommendations based on best available evidence at the time and, when evidence was not available, selected experts from the Neonatal Resuscitation Working Group of the United Nations Commission on Life-Saving Commodities for Women and Children to come to a consensus opinion. These recommendations included steps for preparation, pre-disinfection, high-level disinfection or sterilization, and post-disinfection storage of equipment until next use. Second edition materials that support the new recommendations include a new job aid that presents the steps in a pictorial fashion^48^ and the action plan that specifically recommends that providers “[d]isinfect [equipment] immediately after use” (Figure 2).

### Educational Effectiveness

As a result of the studies showing that ongoing practice was required to retain resuscitation skills, the second edition of the HBB curriculum extends the educational scope of the program beyond a single training to a system of ongoing practice with peer/near-peer support in order to empower providers to change behavior. Birth attendants articulated the need to be equipped with not only technical skills but also a way of reflecting on their own actions and interacting with peers to improve performance.^32^ A page in the second edition facilitator flip chart is devoted to a discussion of devising a system of ongoing practice after an initial HBB workshop (Supplement).^7^ Given the positive effect of debriefings and case reviews after difficult resuscitations, these activities were incorporated as advice toward building the system of ongoing practice. Developing this kind of system was also tied to the concept of supportive supervision that HBB facilitators must provide after the conclusion of an HBB workshop. Low-dose high-frequency practice, associated with decreases in early neonatal and stillbirth mortality, is encouraged. New facilitators are empowered to mentor their learners and leadership at health facilities to oversee the establishment of a system for practice and to supervise peer-to-peer support during practice. Stronger advice is provided for facilitators for this new expanded role.

Further revision of the OSCEs included adding 5 questions that prompt HBB provider to self-reflect on their performance, formulate a plan for improving their behaviors or practices for the next resuscitation, and receive feedback from peers or facilitators. In this way, the OSCEs became more learner-centered and could be used as both a summative and formative evaluation of performance.^49^ These questions also serve the same purpose when used after actual resuscitations.

### Implementation and Sustainability

Given the broader emphasis on implementation, quality improvement, and linkages to existing health care systems as well as the expanded role of facilitators to mentor and oversee these actions, the second edition provides additional advice for the facilitator. Each page in the facilitator flip chart has background information and educational advice to guide the facilitator in techniques for active learning.^7^ The new flip chart emphasizes local implementation with a focus on what the facilitator needs to know and do before, during, and after a course (Supplement), provides a timeline of actions a facilitator should perform when planning a course, and strongly encourages facilitators to integrate their workshops within local health care programs as they plan a training-of-trainers cascade. The flip chart stresses the importance of building and maintaining good relationships with existing in-country programs that already are working toward reducing newborn deaths, as they will be crucial to achieving a broader coverage of skilled birth attendance and sustained implementation. Furthermore, the flip chart serves as a guide to better support facilitators, who now find themselves in a potentially expanded role, not only enabling learning but also facilitating linkages with clinic leadership for ongoing practice and quality improvement efforts within health facilities.

The flip chart emphasizes that facilitators should enable learning but also facilitate linkages with clinic leadership for ongoing practice and quality improvement.

Anecdotally, a typical HBB course concludes with much enthusiasm about the new concepts learned and how these will be implemented in the workshop participant's home facility. Two pages in the second edition were designed to harness and channel provider enthusiasm into specific steps they can incorporate into their facility in order to improve care. A page entitled “Commit to making a difference” uses the revised action plan to point out potential process and outcome indicators that can be used to track improvement (Supplement). It was designed to introduce the process of quality improvement and link neonatal resuscitation to institutional or health system improvement initiatives, without using the daunting jargon that typically accompanies implementation science. The participants are challenged with 3 questions: “What are you going to do differently?” “What will you no longer do?” “How are you going to make these changes happen?” Finally, the flip chart includes a small group exercise where participants can review the information they record on each baby born in their facility, identify potential steps they can take to improve care, and gain insight into how to track whether the changes were successful.

To further aid in accessibility, the teaching materials—in multiple translations—are available online and freely downloadable at hbs.aap.org. Recognizing the difficulty in obtaining and/or printing new batches of teaching materials, the AAP has made available advice on how local providers can adapt their first edition HBB materials to teach the updated concepts. Given the expanded role of facilitators, the AAP created a webinar (https://www.aap.org/en-us/advocacy-and-policy/aap-health-initiatives/helping-babies-survive/Pages/Webinars.aspx) and Global Health Media Project (globalhealthedia.org) produced videos to further promote, educate, and support ongoing mentorship and oversight for systems of practice, quality improvement, implementation, and updated resuscitation practices. Furthermore, AAP and Jhpiego collaborated on a set of implementation briefs that discuss the guiding principles for implementing the Helping Mothers Survive and Helping Babies Survive programs together in a twinned approach, including a focus on competency, simulation, and case-based learning, appropriately spaced brief periods of content delivery, team-focused and facility-based training, ongoing practice of skills after initial training, peer facilitation of practice, results tracking, and comprehensive quality improvement efforts to change service delivery.^50^

### Delphi Review and Field Trials

Given that frontline birth attendants often care for both the mother and the newborn, Delphi reviewers called for better integration of care between the mother and infant. The second edition action plan (Figure 2) explicitly acknowledges that maternal and neonatal care are part of the same sequence by including references to the Helping Mothers Survive program and to preparing oxytocin in the “Prepare for birth” section.

The India field trial, which occurred at the All India Institute of Medical Sciences in New Delhi in June 2016, involved a group of 6 participants with prior training in the first edition HBB curriculum and 18 novice participants. Asking the 3 questions about the “Commit to making a difference” page solicited many answers to identify gaps in care that could be improved. Of the 24 participants, 22 felt well-prepared to be a facilitator after the course. Thematic analysis from qualitative interviews from 3 focus group discussions revealed several strengths of the revised curriculum, with the most effective key themes being the interaction with facilitators, workshop structure, quality improvement, and course content (Table). Participants liked the course emphasis on hands-on learning, rather than lectures, and felt that the small group size allowed participants more time to practice skills and observe and learn from each other's mistakes. They noted that small groups allowed for more personalized interaction with the facilitator, who then was able to give each participant genuine feedback, and felt this made a huge difference in the uptake of the material. The participants commented that the facilitators were able to handle multiple groups at once, provoke discussion, and receive constructive feedback. Further strengths included the new addition of instruction on quality improvement, which now challenged participants to reflect on their own practices and consider how they could improve. They found the quality improvement material empowered individuals to see what they could do to bring change to their facility. Even so, participants were concerned that some individuals in peripheral clinics may not see the value in quality improvement, because quality was perceived as a luxury that was only for places that have the resources to effect change. Participants felt that the content was presented and displayed in a clear and concise manner, and that the flip chart contained more guidance for facilitation and more instruction on how to organize and conduct a workshop.

**Figure:**
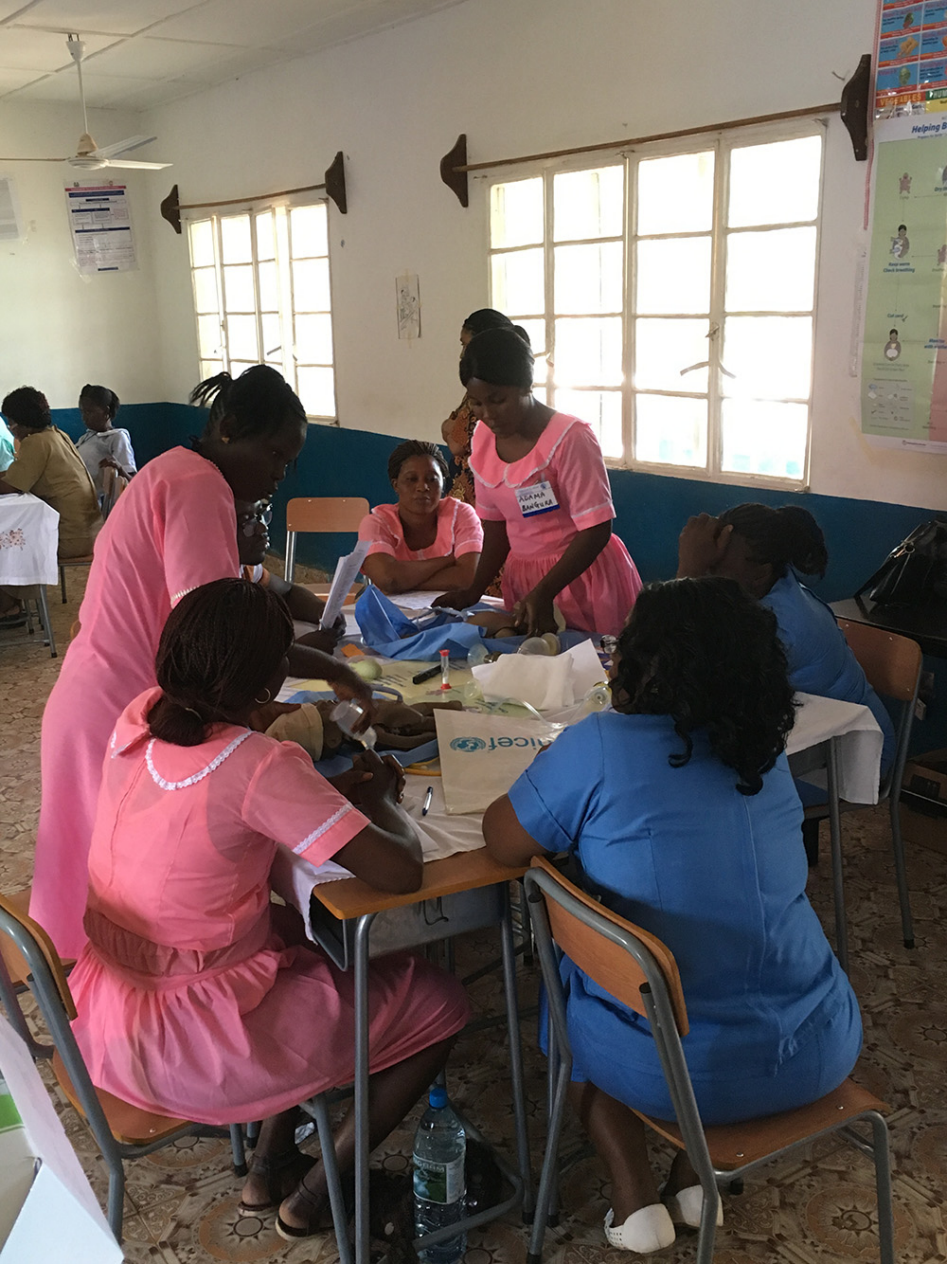
Field testing of Helping Babies Breathe 2nd Edition with MNCH aides in Kabala, Sierra Leone. © 2016 Erick Amick/American Academy of Pediatrics.

**TABLE.** Key Training Themes in Qualitative Analysis From Helping Babies Breathe Second Edition Field Testing in India

Themes	Subthemes	Selected Comments
**Most Effective Themes**		
Facilitators	Provoke discussion Feedback One-on-one interaction	“The facilitator could give feedback, genuine feedback.”
Workshop structure	Emphasis on doing rather than lecturing Small group size Time for practice, observation, and learning from each other's mistakes Enough equipment for everyone to practice with Networking with others outside of their area to discuss their practice similarities and differences	“I think it uses everybody's time more effectively. I think working in small groups … that was the beauty of the program.”
Quality improvement	Reflection of each individual's practice and how to improve Worked well with rest of workshop structure	“[B]ecause we train so many people, and we impart knowledge and skills, and it is individual improvement which is looked at. That session for the first time, looked at the individual and what he/she will do to bring change to their unit.”
Content	Clear and concise presentation of information Action plan was a helpful summary Flip chart serving as a written guide for facilitating	“Planning of the flip chart and implementation guide has been incorporated. That was the one thing which everyone wanted. While you are organizing and conducting a workshop, what you need to do, and what our facilitators should do, is now written.”
**Least Effective Themes**		
Content	Suggestive or ambiguous language such as “may” or “could” Time to read and review material prior to the course Complexity of content for peripheral clinics	“If it is meant mostly for peripheral settings, where resources are scarce, and I think the messages have to be direct and loud, that message is not obvious. Because you can say from this what is most important? It doesn't strike.”
Format	Presentation of material (color coding, font size, binding of flip chart)	
Integration	How to ensure implementation at facility after training Skills would not be maintained unless practiced and refresher courses available Administrative support of the program at their facility Buy-in from local leaders	“Otherwise we go through the same cycle of doing the workshop, but having no impact.” “There has to be some time, some kind of timeline, that every day or alternate day, or once a week they have a practice session of this duration. And the unit in charge should be made responsible for this action.”
Unrealistic expectations	Difficult to implement with low resources Supply chain Facility where workshop held versus reality in periphery Overemphasis of skin-to-skin care Concerns about resources and space Lack of experience with quality improvement	“[How to do skin-to-skin care] is all very vague, and therefore it may not be taken up well. So more clarity on that and the pictures need to be done.” “Quality is considered a very special thing. It is something that is a luxury for people who have a lot of resources. This is their mindset. Because in a source limited situation, quality cannot be done. This is the mindset. So how to change that mindset?”

Of the 24 participants in the India field trial, 22 felt well-prepared to be a facilitator after the course.

The Sierra Leone field trial occurred in August 2016 at Kabala District Health Management Facility in Koinadugu district and consisted of 24 participants from nearby facilities with no prior exposure to the HBB curriculum. Participants were professionally diverse and included midwives, public health nurses, hospital matrons, a community health officer, and maternal and neonatal health aides. The training was conducted in 2 stages, with 12 participants learning the HBB curriculum in each stage. Four participants in the first stage were selected to become master trainers, who subsequently trained the remaining 12 participants in the second stage. Following the trainings, written evaluations were administered and participants were invited to join focus group discussions. Written evaluations demonstrated that the participants valued hands-on practice, small-group discussion, and interaction with the facilitators. In focus group discussions, participants noted that certain common practices would be reevaluated, such as routine suctioning and emphasis on resuscitation with chest compressions. New master trainers felt comfortable using the materials to impart the knowledge and skills to their learners, but they felt that giving feedback was a skill they needed to improve, and they noted that their learners needed to become accustomed to feedback as part of the learning process. Observations of AAP master trainers showed that these new master trainers from Sierra Leone reverted back to lecturing rather than facilitating discussion about the newly included topics of quality improvement and “what the facilitator needs to know and do.” The new master trainers themselves noted that more time was needed after the initial HBB workshop to better understand these concepts.

New master trainers felt that giving feedback was a skill they needed to improve and something learners needed to become accustomed to.

### Final Revisions

After the field trials, the inputs were all critically reviewed by program leadership. Careful language had to be chosen to convey the role of suctioning and to stimulate discussion and an evidence review regarding equipment reprocessing. While the quality improvement content was well received and the ideas and energy toward quality improvement were self-initiated after workshop, the concepts needed to be further simplified and further coaching was considered beneficial to help accelerate the work. Additional input from global leaders and implementers was used to strengthen the recommended advice for facilitators before, during, and after the workshop to make linkages with existing health care systems, plan for systems of ongoing practice, and engage in quality improvement.

## DISCUSSION

The global community must remain committed to introducing practices that will accelerate the reduction of overall neonatal mortality in order to meet the goals of the Every Newborn Action Plan.^21^ These goals include achieving national neonatal mortality rates of less than 10 neonatal deaths per 1,000 live births with the aim of achieving global neonatal mortality rates of less than 7 neonatal deaths per 1,000 live births, all by 2035.^21^ Despite all efforts to decrease neonatal mortality, recent data show that neonatal mortality has declined at a slower rate than overall childhood mortality, which has resulted in neonatal mortality now accounting for 46% of overall under-5 childhood deaths.^51^

Neonatal resuscitation is an important intervention with the potential to save newborn lives. HBB addresses not only the science of resuscitation but also the key steps to improve educational efficiency and health care delivery that are essential to improving neonatal survival. HBB has been combined with the Essential Care for Every Baby, Essential Care for Small Babies, and Improving Care for Mothers and Babies curricula to create a suite of Helping Babies Survive programs.^52^^–^^54^ The Helping Babies Survive programs use similar effective educational approaches that emphasize facilitated learning and promote continued practice and quality improvement to further enhance survival and decrease neonatal morbidity.^52^^–^^54^

By combining HBB, Essential Care for Every Baby, Essential Care for Small Babies, and Improving Care for Mothers and Babies curricula, a single suite of Helping Babies Survive programs was created to address the needs of both mothers and babies.

The process for creating the second edition of HBB included reflection that the impact of the program encompassed more than just medical science and extended to the other 2 components of the Utstein Formula for Survival—educational efficacy and local implementation. Since the release of the first edition, gains in newborn survival have been achieved and essential lessons learned regarding the importance of ongoing practice for retention of skills and quality improvement to enhance and ensure evidence-based practices were occurring at the individual and facility levels. Further feedback from frontline users have been incorporated to make the educational program more accessible and provide guidance to facilitators, program managers, and policy makers on the importance of incorporating interventions such as quality improvement and systems of ongoing practice for maintenance of resuscitation skills.

The challenge now is to ensure that the updated science and the concepts of ongoing practice and quality improvement reach all health workers attending deliveries and that the materials continue to be easily accessible to all. Since the release of the first edition of the HBB curriculum in 2010, an impressive number of providers around the world have been trained in the skills of basic neonatal resuscitation. While broad coverage and widespread dissemination are still essential, the second edition emphasizes that continued follow-up, ongoing practice, and quality improvement are critical to improving outcomes and further decreasing neonatal mortality. The AAP remains committed to ensuring the most up-to-date recommendations are available to all users; the AAP hosts the Helping Babies Survive website (hbs.aap.org) where downloads of the updated HBB materials—in addition to the other Helping Babies Survive curricula—are freely available as well as information sheets that describe how to adapt first edition materials to stay current with the most recent recommendations. National health leaders and ministries can use the materials to update national clinical guidelines to reflect the most recent resuscitation science, as these are highlighted on the website.

The expanded role for facilitators also deserves further attention; their efforts at catalyzing behavior change in the areas where they are working begin with facilitating an HBB workshop and continue as they support practitioners to maintain their skills after the workshop is over. HBB facilitators now work with local clinical leadership to establish a system for ongoing mentorship, committed supervision, and policies that support neonatal resuscitation training as an organizational routine. Additional tools to assist facilitators are under development by the AAP Helping Babies Survive Planning Group.

The Helping Babies Survive programs have demystified some of the practices related to newborn care and made them easily accessible to local providers all over the world. This innovative model of education has successfully transmitted current resuscitation science and has expanded to address provider behavior change and delivery system quality improvement. The capacity to change patient outcomes has been demonstrated in both small- and large-scale trials. The programs have achieved many successes, including champions who have created ongoing systems of practices at peripheral facilities, country facilitators who have originated national training-of-trainers cascades, academics and clinicians who have included Helping Babies Survive programs in preservice curricula, nurses and midwives who have been empowered to improve care, and researchers who have created data collection systems to monitor the success or challenges to implementation.

However, in order to truly impact neonatal mortality, additional steps need to be taken to address sustainability in order to make high-quality effective neonatal resuscitation a permanent part of the health system. Achieving impact at the population level will require integration into the national health system, with incorporation of adapted educational materials into comprehensive preservice and in-service education packages, mechanisms for supply and logistics management, and linkages with quality improvement initiatives to effect change and document outcomes. Furthermore, neonatal resuscitation is only one aspect of overall essential newborn care. In order to reduce neonatal mortality, improved essential newborn care and supportive care for small and sick newborns will be crucial. Care of the newborn within the continuum of perinatal care also calls for investments in maternal care to prevent asphyxia and reduce preterm birth and associated morbidities. Ongoing efforts on the part of governments and stakeholders to bring coverage and quality of neonatal resuscitation to scale have the potential to achieve impact on global neonatal mortality.

## Supplementary Material

18-00147-Kamath-Rayne-Supplement.pdf

## Notes

**Peer Reviewed**

**Funding:** Latter-day Saint Charities provided funding for the field trials in India and Sierra Leone.
