# Supplementary material for: Helping Babies Breathe, Second Edition: A Model for Strengthening Educational Programs to Increase Global Newborn Survival
Source: Glob Health Sci Pract. 2018 Oct 3;6(3):538–51. doi: 10.9745/GHSP-D-18-00147 (PMC6172134; doi:10.9745/GHSP-D-18-00147)
Supplement: 18-00147-Kamath-Rayne-Supplement.pdf [file 18-00147-Kamath-Rayne-Supplement.pdf]

Helping Babies Breathe 2nd Edition
Summary of Changes

Helping Babies Breathe (HBB) 2nd Edition includes scientific updates to harmonize with 2015 ILCOR Consensus on Science with Treatment Recommendations, and 2012 WHO Guidelines on Basic Newborn Resuscitation. It also features strengthened educational advice and new guidance on program implementation and quality improvement, based on feedback and lessons learned from the global community of HBB users.

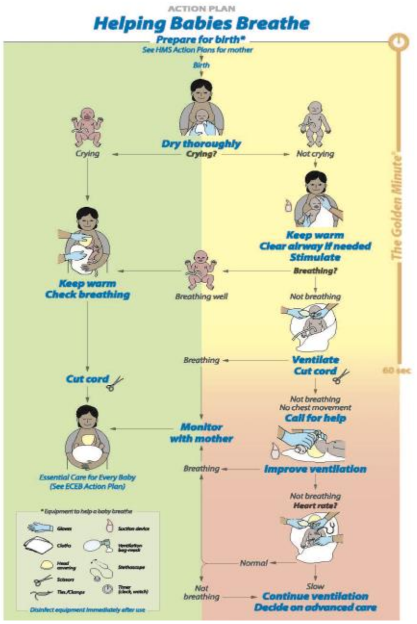

Updates to the HBB Action Plan

- Added link with *Helping Mothers Survive*
  - Text – *"See HMS Action Plan for mother"*
  - Illustration of baby on mother's chest
- Eliminated "if meconium, clear the airway" before the action step, "Dry thoroughly"
- Changed indications for suctioning to be performed if airway is obstructed, or if meconium in amniotic fluid and baby is not crying after thorough drying
- Added option for initiating ventilation
  - Decision to begin ventilation with cord intact or after clamping and cutting the cord
- Added link with *Essential Care for Every Baby*

The Learner Workbook becomes the new Provider Guide

- Clear de-emphasis of suctioning
  - Removal of recommendation for suctioning before thorough drying if meconium in amniotic fluid
  - Articulation of two elements of airway clearing - positioning and suctioning (only if indicated)
  - Emphasis on hazards of prolonged or overly vigorous suctioning
- Added option for delayed cord clamping after initiation of ventilation
- Emphasis on rapid evaluation of chest movement and quick corrective steps after initiation of ventilation
- Emphasis on uninterrupted ventilation until the baby is breathing spontaneously (simplified red zone – continued ventilation with normal or slow heart rate)

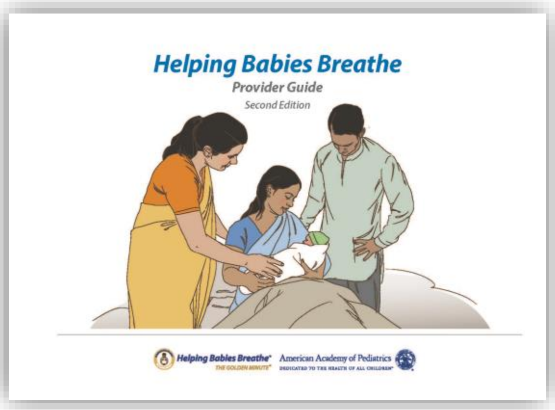

The HBB Facilitator Flip Chart now contains more detailed educational advice and additional resources for implementation and quality improvement

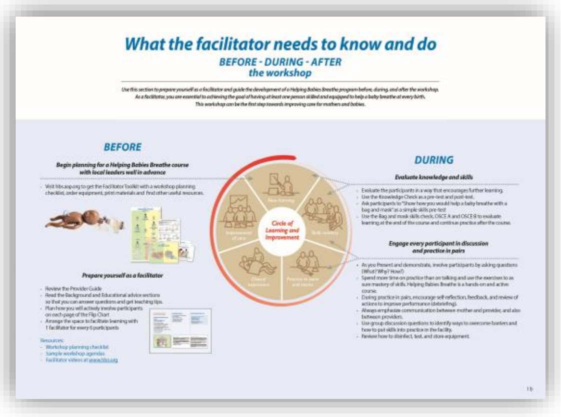

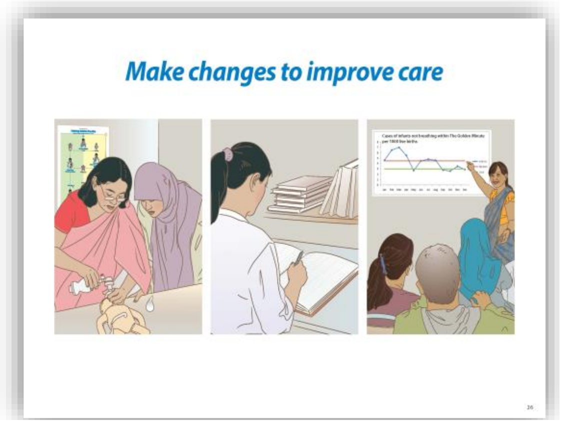

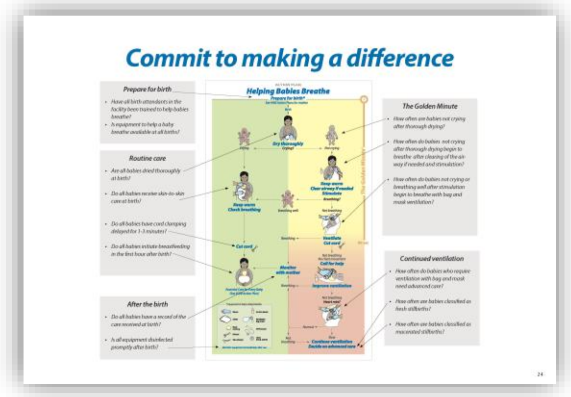

Updates to the HBB Facilitator Flip Chart

- A checklist to guide facilitator before, during, and after the workshop
- More specific educational advice to accompany skills
  - Additional segment on implementation
  - Commit to making a difference
  - In-facility routines for practice and mentoring
  - Introduction to quality improvement
- Emphasis on implementation of resuscitation in a facility
- Low-dose, high frequency practice; self-reflection, debriefing, and case reviews; quality improvement
- Annotated Action Plan with suggested quality measures
- Revised advice on disinfection of equipment to follow *Reprocessing Guidelines for Basic Neonatal Resuscitation Equipment in Resource-limited Settings*

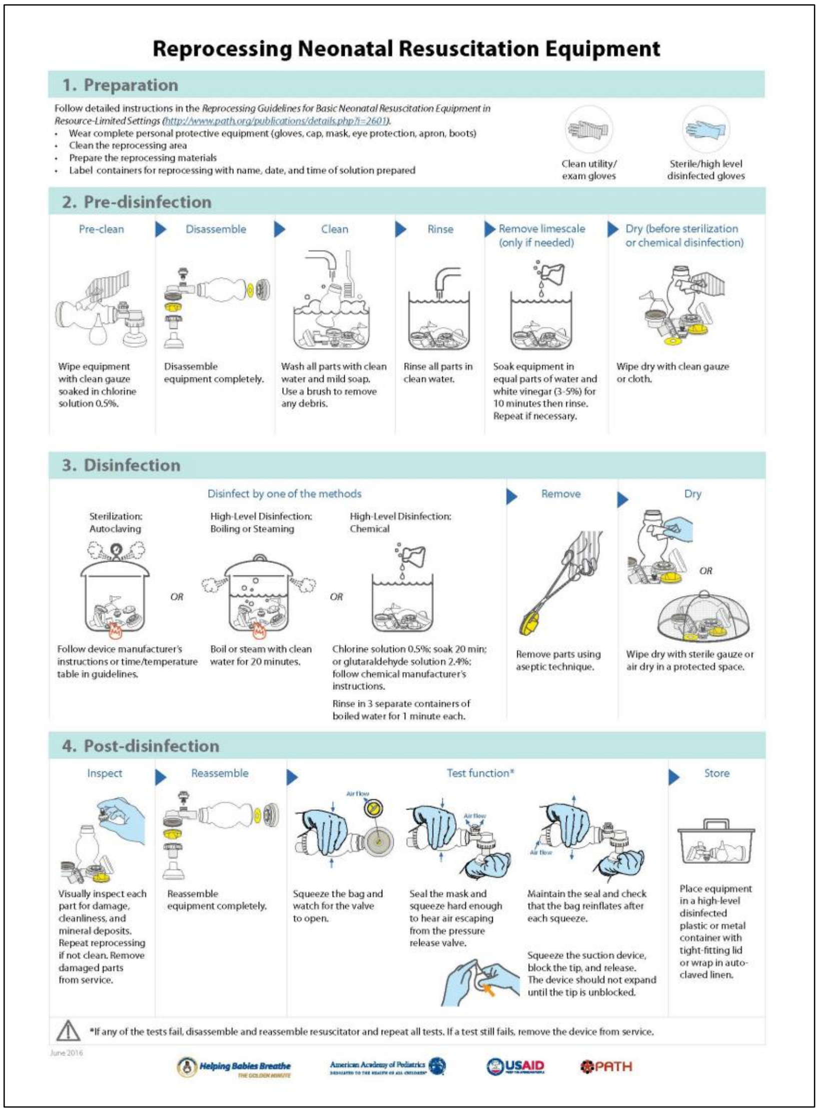

Reprocessing Neonatal Resuscitation Equipment

1. Preparation

Follow detailed instructions in the *Reprocessing Guidelines for Basic Neonatal Resuscitation Equipment in Resource-Limited Settings* (http://www.path.org/publications/details.php?i=2601).

- Wear complete personal protective equipment (gloves, cap, mask, eye protection, apron, boots)
- Clean the reprocessing area
- Prepare the reprocessing materials
- Label containers for reprocessing with name, date, and time of solution prepared

Clean utility/ exam gloves

Sterile/high level disinfected gloves

2. Pre-disinfection

Pre-clean — Wipe equipment with clean gauze soaked in chlorine solution 0.5%.

Disassemble — Disassemble equipment completely.

Clean — Wash all parts with clean water and mild soap. Use a brush to remove any debris.

Rinse — Rinse all parts in clean water.

Remove limescale (only if needed) — Soak equipment in equal parts of water and white vinegar (3-5%) for 10 minutes then rinse. Repeat if necessary.

Dry (before sterilization or chemical disinfection) — Wipe dry with clean gauze or cloth.

3. Disinfection

Disinfect by one of the methods

Sterilization: Autoclaving — Follow device manufacturer's instructions or time/temperature table in guidelines.

OR

High-Level Disinfection: Boiling or Steaming — Boil or steam with clean water for 20 minutes.

OR

High-Level Disinfection: Chemical — Chlorine solution 0.5%: soak 20 min; or glutaraldehyde solution 2.4%: follow chemical manufacturer's instructions.

Rinse in 3 separate containers of boiled water for 1 minute each.

Remove — Remove parts using aseptic technique.

Dry — Wipe dry with sterile gauze or air dry in a protected space. (OR)

4. Post-disinfection

Inspect — Visually inspect each part for damage, cleanliness, and mineral deposits. Repeat reprocessing if not clean. Remove damaged parts from service.

Reassemble — Reassemble equipment completely.

Test function* — Air flow

Squeeze the bag and watch for the valve to open.

Seal the mask and squeeze hard enough to hear air escaping from the pressure release valve.

Maintain the seal and check that the bag reinflates after each squeeze.

Squeeze the suction device, block the tip, and release. The device should not expand until the tip is unblocked.

Store — Place equipment in a high-level disinfected plastic or metal container with tight-fitting lid or wrap in auto-claved linen.

*If any of the tests fail, disassemble and reassemble resuscitator and repeat all tests. If a test still fails, remove the device from service.

June 2016

Helping Babies Breathe THE GOLDEN MINUTE · American Academy of Pediatrics · USAID · PATH

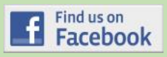

HelpingBabiesSurvive

follow us on twitter

@AAPGlobalHealth

Browse our Website
http://hbs.aap.org

Email us
hsb@aap.org
